# Supplementary material for: Understanding the hemodynamic changes in fetuses with coarctation of the aorta using a lumped model of fetal circulation
Source: PLoS Comput Biol. 2025 May 30;21(5):e1013096. doi: 10.1371/journal.pcbi.1013096 (PMC12124859; doi:10.1371/journal.pcbi.1013096)
Supplement: S1 Appendix — (DOCX) [file pcbi.1013096.s001.docx]

**Validation of our physiological regions considering variations in ventricular depth**

We validated our physiological regions with ultrasonographic measurements from 7 controls and 9 CoA patients. For computing the right/left ventricular end-diastolic volume (RV/LV EDV) ratio, we assumed that the change in ventricular depth was similar for both ventricles. We further validated these regions by considering depth changes proportional to the square of the ventricular area variation, applying the formula RV/LV EDV = (RV area)^3/2^/(LV area)^3/2^. As shown in S1 Fig, most real-case data points fall within or very close to the physiological regions, further supporting their validity. Only one case deviated slightly, likely due to uncertainties in our model parameters (see S3 Fig).

**Scaling of the model’s parameters according to gestational age**

We adjusted all parameters representing the vasculature of the reference healthy model to obtain pressures, blood flows, and velocities within a physiological range for a fetus of 32 weeks of gestational age (GA), using equations described by Pennati and Fumero[1] and Gallivan *et al.*[2]. Any parameter ($X$) is assumed to be proportional to body weight ($W$) with a scaling factor $b$. Therefore, at two different time points ($GA_{1}$, $GA_{2}$), with respective weights ($W_{1}$, $W_{2}$), the parameter satisfies the following equation[1]:

$$X\left( GA_{1} \right)=X\left( GA_{2} \right)\cdot\left( \frac{W_{1}}{W_{2}} \right)^{b}.$$

Moreover, according to Gallivan *et al.*[2], $W$ (in grams) and $GA$ (in weeks) in healthy human fetuses are related by

$$\log_{10} W=0.2508+0.1458\cdot GA-0.0016\cdot GA^{2}.$$

With these formulas, we used the values of all vasculature parameters reported by Pennati *et al.*[3] at 38 weeks of GA to compute those at 32. For 38 weeks of GA, according to Gallivan *et al.*[2] the corresponding healthy weight is 3025.5 grams, while for 32 weeks the weight is 1896.7 grams. Therefore, any parameter at 32 weeks can be computed from that at 38 as

$$X\left( 32 \right)=X\left( 38 \right)\cdot{0.6269}^{b}.$$

We set the scaling factor $b$ according to the values reported by Pennati and Fumero[1]. Since the diameters and lengths of vessels can be described by allometric equations using scaling factors close to 0.33[4], $b$ was set to -1 for resistances, -0.33 for inductances, and 1.33 for capacitances. Due to their unusual diameter growth, peculiar locations, and functions in the fetal cardiovascular system[1], the scaling factors of the non-linear resistances in ductus venosus, ductus arteriosus, and foramen ovale were set to -0.88, -2.5, and -0.6, respectively.

**Greatest variation in literature of cardiac parameters for CoA**

We extracted from the literature the expected greatest variation of the different cardiac parameters in coarctation of the aorta (CoA) with respect to control populations. We obtained the minimum aortic isthmus (AoI) diameter[5–9] and the maximum ductus arteriosus (DA) diameter[6, 9] reported for CoA, which we considered to be present in the most severe CoA, and their respective mean values reported for control populations. Whenever a z-score was provided, we computed the corresponding value for a GA of 32 weeks using the z-score conversion formula[10, 11], under the assumption that the z-score remains stable across gestation[7]. To account for the difference between the reported GA and EFW with those of our model, for each variable we computed the ratio between the most abnormal value reported in the literature and the controls’ mean value. All these data are collected in S1 Table for AoI and S2 Table for DA. For ventricular disproportion, as the RV/LV end-diastolic volume (EDV) ratio was not found to be documented, we used the most severe RV/LV mid-transverse diameter ratio reported in Soveral *et al.*[12] (210%), as other cardiac dimensions in CoA remained similar to those in healthy cases[12].

**Description of ventricular disproportion implementation**

To simulate the effect of ventricular disproportion, we increased the RV/LV EDV ratio. However, this variable cannot be directly accessed in our model since it is an output rather than an input, so we changed the myocardial mass volume (Vwall) of the LV instead and then increased the RV Vwall. Since clinical studies have not reported that combined cardiac output is reduced in fetuses with CoA[12], we fixed a certain value for LV Vwall (from 100% to 30% of the original value) and estimated the optimal RV Vwall by minimizing the difference between the combined cardiac output of the ventricular disproportion and that of the control simulation. Similarly, as ventricular hypertrophy has not been reported in CoA either[12], we estimated the left and right ventricular reference areas (Amref, i. e. the wall area at zero pressure) so that the ratio of Vwall to cavity volume was preserved in both LV and RV. To estimate all these parameters, we used Brent’s optimization method[13].

We then decreased the left atrial Vwall and Amref with the same factors as those of the LV. The mitral and aortic valves orifice areas and the ascending aorta radius were also shrunk with 2/3 and 1/3 factor of the LV Vwall reduction, respectively. Their corresponding resistances (R), capacitances (C) and/or inductances (L) were recalculated as a function of the resulting radius $r$ assuming $R\propto r^{-4}$, $C\propto r^{2}$ and $L\propto r^{-2}$ [14]. In a similar way, the right atrium, tricuspid and pulmonary valves, and main pulmonary artery were dilated according to the RV enlargement.

**Uncertainty analysis on the model’s parameters**

To increase the reliability of our predictions, we conducted an uncertainty analysis. We derived 10 different parameter sets from the original parameters by introducing random variations of up to 10%, and generated all CoA scenarios. For each case, we defined its physiological regions - combinations of aortic isthmus (AoI) narrowing, right/left ventricular end-diastolic volume (RV/LV EDV) ratio, and ductus arteriosus (DA) dilation with changes below 5%, 10%, and 15% across all anatomical sites. We then calculated the 25% (blue) and 75% (orange) confidence interval regions, as shown in S3 Fig, which closely surrounded or fell within our original regions. This further supports our conclusion that the co-occurrence of ventricular disproportion and DA dilation is essential to compensate for the coarctation.

**Bibliography**

1. Pennati G, Fumero R (2000) Scaling approach to study the changes through the gestation of human fetal cardiac and circulatory behaviors. Ann Biomed Eng 28:442–452

2. Gallivan S, Robson SC, Chang TC, Vaughan J, Spencer JAD (1993) An investigation of fetal growth using serial ultrasound data. Ultrasound in Obstetrics and Gynecology 3:109–114

3. Pennati G, Bellotti M, Fumero R (1997) Mathematical modelling of the human foetal cardiovascular system based on Doppler ultrasound data. Med Eng Phys 19:327–335

4. Holt JP, Rhode EA, Holt WW, Kines H Geometric similarity of aorta, venae cavae, and certain of their branches in mammals.

5. Beattie M, Peyvandi S, Ganesan S, Moon-Grady A (2017) Toward Improving the Fetal Diagnosis of Coarctation of the Aorta. Pediatr Cardiol 38:344–352

6. Fricke K, Liuba P, Weismann CG (2021) Fetal Echocardiographic Dimension Indices: Important Predictors of Postnatal Coarctation. Pediatr Cardiol 42:517–525

7. Xu R, Zhou D, Liu Y, Yao L, Xie L, Liu M, Zhou Q, Zeng S (2023) Impaired Elastic Properties of the Ascending Aorta in Fetuses With Coarctation of the Aorta. J Am Heart Assoc. https://doi.org/10.1161/JAHA.122.028015

8. Contro E, Cattani L, Balducci A, et al (2022) Prediction of neonatal coarctation of the aorta at fetal echocardiography: a scoring system. Journal of Maternal-Fetal and Neonatal Medicine 35:4299–4305

9. Gomez-Montes E, Herraiz I, Mendoza A, Escribano D, Galindo A (2013) Prediction of coarctation of the aorta in the second half of pregnancy. Ultrasound in Obstetrics and Gynecology 41:298–305

10. Schneider C, McCrindle BW, Carvalho JS, Hornberger LK, McCarthy KP, Daubeney PEF (2005) Development of Z-scores for fetal cardiac dimensions from echocardiography. Ultrasound in Obstetrics and Gynecology 26:599–605

11. Pasquini L, Mellander M, Seale A, Matsui H, Roughton M, Ho SY, Gardiner HM (2007) Z-scores of the fetal aortic isthmus and duct: An aid to assessing arch hypoplasia. Ultrasound in Obstetrics and Gynecology 29:628–633

12. Soveral I, Crispi F, Walter C, et al (2020) Early cardiac remodeling in aortic coarctation: insights from fetal and neonatal functional and structural assessment. Ultrasound in Obstetrics and Gynecology 56:837–849

13. Brent RP. (2013) Algorithms for Minimization Without Derivatives. Courier Corporation

14. Garcia-Canadilla P, Rudenick PA, Crispi F, Cruz-Lemini M, Palau G, Camara O, Gratacos E, Bijens BH (2014) A Computational Model of the Fetal Circulation to Quantify Blood Redistribution in Intrauterine Growth Restriction. PLoS Comput Biol. https://doi.org/10.1371/journal.pcbi.1003667
