# Supplementary material for: Understanding the hemodynamic changes in fetuses with coarctation of the aorta using a lumped model of fetal circulation
Source: PLoS Comput Biol. 2025 May 30;21(5):e1013096. doi: 10.1371/journal.pcbi.1013096 (PMC12124859; doi:10.1371/journal.pcbi.1013096)
Supplement: S1 Table — (DOCX) [file pcbi.1013096.s004.docx]

**S1 Table**. Aortic isthmus diameter reported in different clinical studies and expected greatest variation in coarctation of the aorta with respect to control populations.

| **Study** | **Controls (mm)** | **CoA (mm)** |
| --- | --- | --- |
| Beattie et al.[1] | 2.844 (2.526-3.201) | 2.383 (1.892-3.003) |
| Fricke et al.[2] | 3.23 (2.792-3.538) | 2.504 (1.821-2.767) |
| Xu et al.[3] | 3.925 (2.896-4.999) | 1.834 (1.288-2.436) |
| Contro et al.[4] | 2.742 (1.94-3.875) | 2.164 (1.772-2.644) |
| Gomez-Montes et al.[5] | 3.058 (2.596-3.603) | 2.55 (1.94-3.35) |
| Mean | 3.160 (1.940-4.999) | 2.287 (1.288-3.35) |
| Greatest variation | - | 40.76 % |

Data are expressed as median (interquartile range).

**Bibliography**

1. Beattie M, Peyvandi S, Ganesan S, Moon-Grady A (2017) Toward Improving the Fetal Diagnosis of Coarctation of the Aorta. Pediatr Cardiol 38:344–352

2. Fricke K, Liuba P, Weismann CG (2021) Fetal Echocardiographic Dimension Indices: Important Predictors of Postnatal Coarctation. Pediatr Cardiol 42:517–525

3. Xu R, Zhou D, Liu Y, Yao L, Xie L, Liu M, Zhou Q, Zeng S (2023) Impaired Elastic Properties of the Ascending Aorta in Fetuses With Coarctation of the Aorta. J Am Heart Assoc. https://doi.org/10.1161/JAHA.122.028015

4. Contro E, Cattani L, Balducci A, et al (2022) Prediction of neonatal coarctation of the aorta at fetal echocardiography: a scoring system. Journal of Maternal-Fetal and Neonatal Medicine 35:4299–4305

5. Gomez-Montes E, Herraiz I, Mendoza A, Escribano D, Galindo A (2013) Prediction of coarctation of the aorta in the second half of pregnancy. Ultrasound in Obstetrics and Gynecology 41:298–305
