# Supplementary material for: Understanding the hemodynamic changes in fetuses with coarctation of the aorta using a lumped model of fetal circulation
Source: PLoS Comput Biol. 2025 May 30;21(5):e1013096. doi: 10.1371/journal.pcbi.1013096 (PMC12124859; doi:10.1371/journal.pcbi.1013096)
Supplement: S2 Table — (DOCX) [file pcbi.1013096.s005.docx]

**S2 Table**. Ductus arteriosus diameter reported in different clinical studies and expected greatest variation in coarctation of the aorta with respect to control populations.

| **Study** | **Controls (mm)** | **CoA (mm)** |
| --- | --- | --- |
| Fricke et al.[6] | 4.587 (3.949-5.327) | 5.234 (4.444-5.796) |
| Gomez-Montes et al.[9] | 4.709 (3.492-6.352) | 5.421 (4.709-6.241) |
| Mean | 4.648 (3.492-6.352) | 5.328 (4.444-6.241) |
| Greatest variation | - | 134.27 % |

Data are expressed as median (interquartile range).
