# Supplementary figures and images for: Understanding the hemodynamic changes in fetuses with coarctation of the aorta using a lumped model of fetal circulation
Source: PLoS Comput Biol. 2025 May 30;21(5):e1013096. doi: 10.1371/journal.pcbi.1013096 (PMC12124859; doi:10.1371/journal.pcbi.1013096)

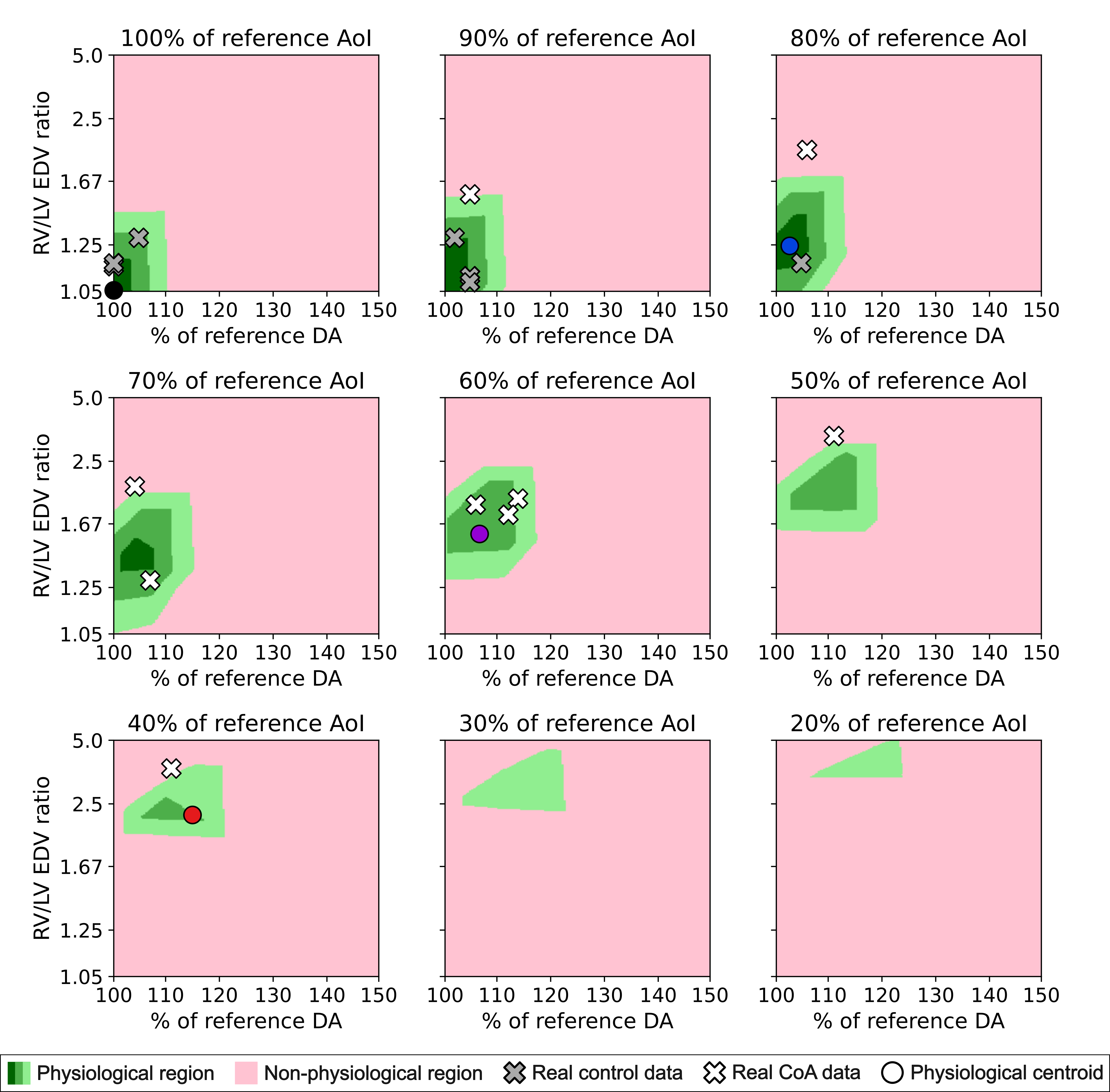

Supplement: S1 Fig — Distribution of regions in which total blood flow, wall shear stress and pressures are below a 5% (dark green), 10% (medium green), and 15% (light green) change in the whole fetal body relative to the healthy values, or exceed that amount at any anatomical site (pink), for different combinations of aortic isthmus (AoI) narrowing, right-to-left ventricular end-diastolic volume (RV/LV EDV) ratio and ductus arteriosus (DA) dilation. Real healthy and coarctation of the aorta (CoA) cases are marked with grey and white crosses, respectively, where ventricular depth is assumed to change proportionally to the ventricular area. Points in which the percentage of change of the physiological region with respect to the healthy reference model is minimal are marked with grey, blue, purple, and red circles for 100%, 80%, 60%, and 40% of the reference aortic isthmus size, respectively. (TIFF) [file pcbi.1013096.s006.tiff]

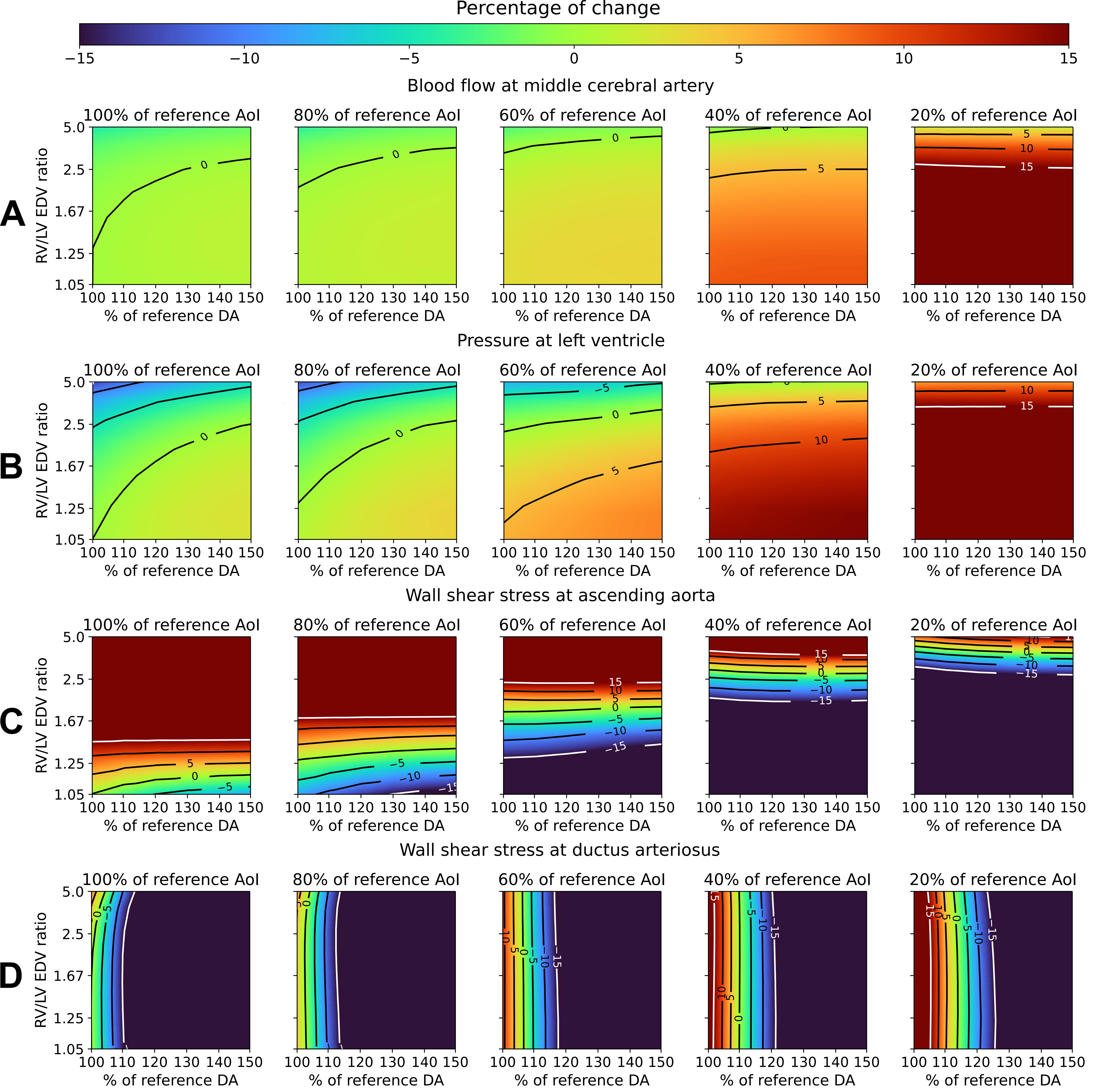

Supplement: S2 Fig — Percentages of change of: total blood flow through (A) the middle cerebral artery and (B) the umbilical artery; wall shear stress at (C) ascending aorta, (D) main pulmonary artery, (E) ductus arteriosus (DA); and (F) pressure at the left ventricle for different combinations of aortic isthmus (AoI) narrowing, right-to-left ventricular end-diastolic volume (EDV) ratio and DA dilation. Isolines indicate absolute change percentages of 0%, 5%, 10% and 15%. (TIFF) [file pcbi.1013096.s007.tiff]

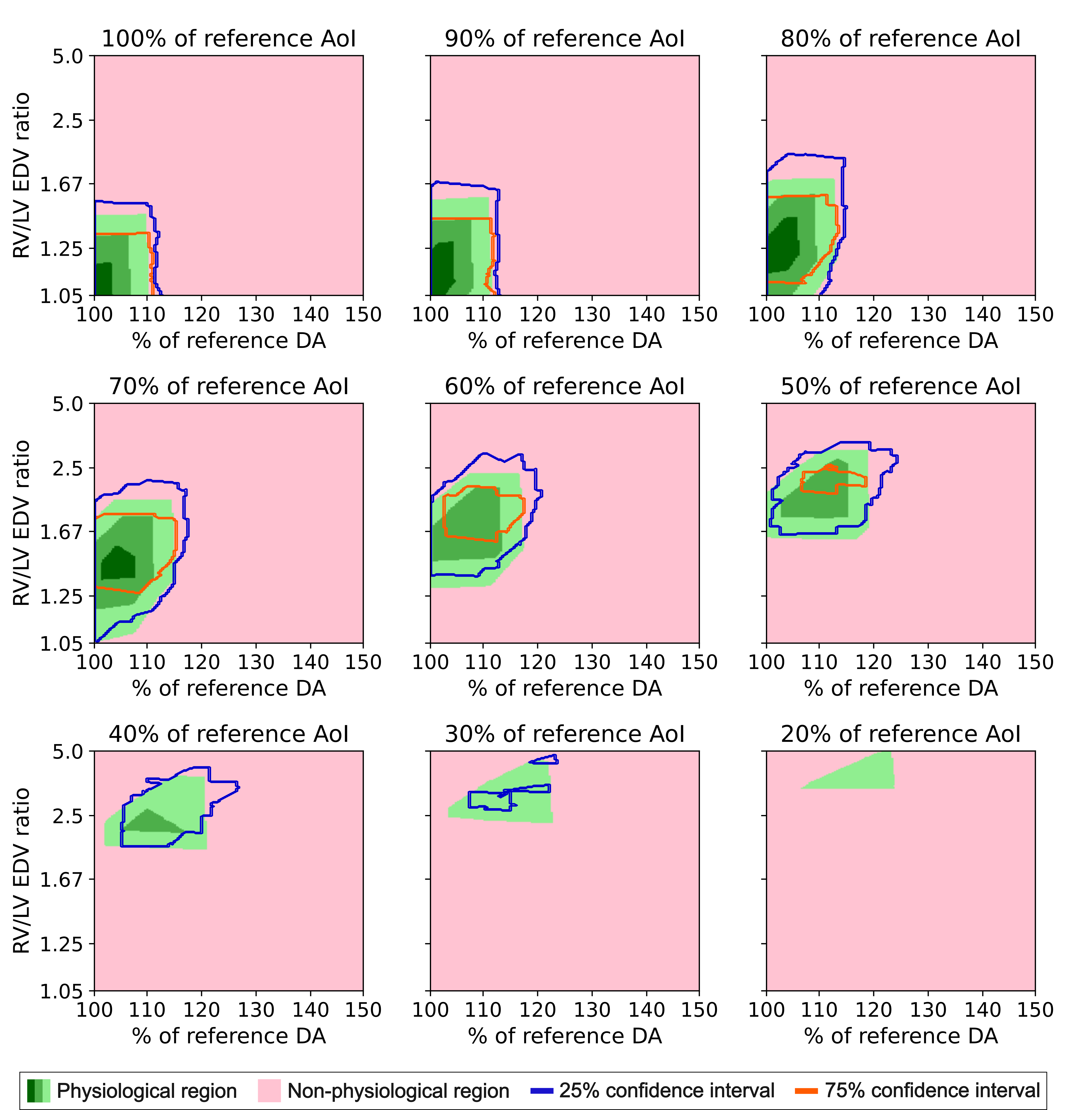

Supplement: S3 Fig — Distribution of regions in which total blood flow, wall shear stress and pressures are below a 5% (dark green), 10% (medium green), and 15% (light green) change in the whole fetal body relative to the healthy values, or exceed that amount at any anatomical site (pink), for different combinations of aortic isthmus (AoI) narrowing, right-to-left ventricular end-diastolic volume (RV/LV EDV) ratio and ductus arteriosus (DA) dilation. 25% (blue) and 75% (orange) confidence interval areas are also depicted. (TIFF) [file pcbi.1013096.s008.tiff]

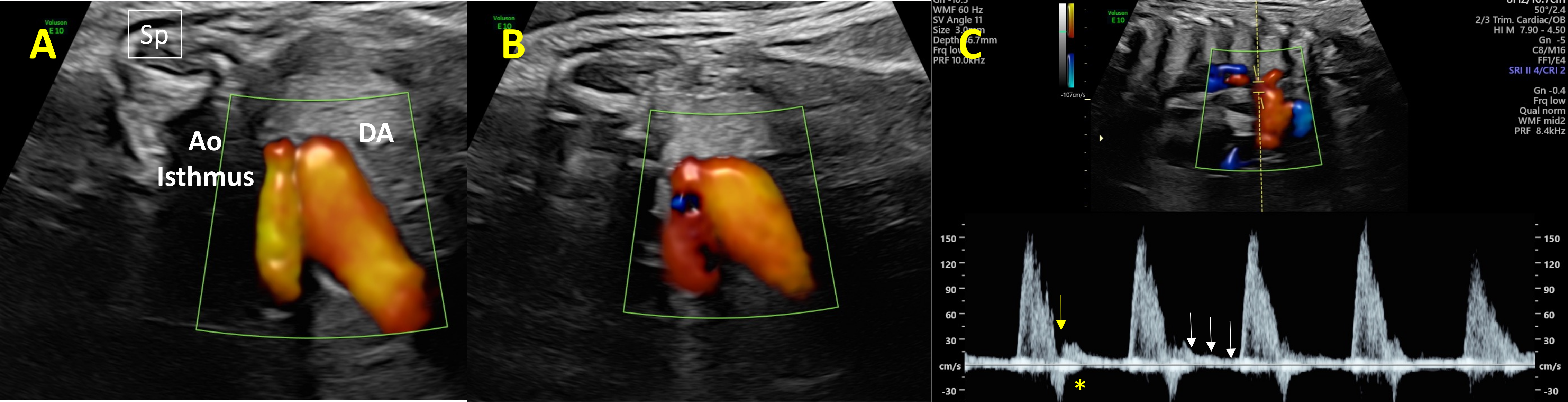

Supplement: S4 Fig — (A) The 3-vessel and trachea view evaluated with conventional color Doppler shows synchronic anterograde flow in the transverse aortic arch and aortic isthmus compared with the main pulmonary artery and ductus arteriosus. (B) A small amount of reversed flow is evident at the end of the systole (codified in blue). (C) Aortic isthmus flow characteristics by spectral pulsed-Doppler. Note the presence of a late-systolic notch (yellow arrow) with a small reversal flow wave (*) and very low flow rate in diastole (white arrows). Ao: aorta, DA: ductus arteriosus, Sp: spine. (TIFF) [file pcbi.1013096.s009.tiff]

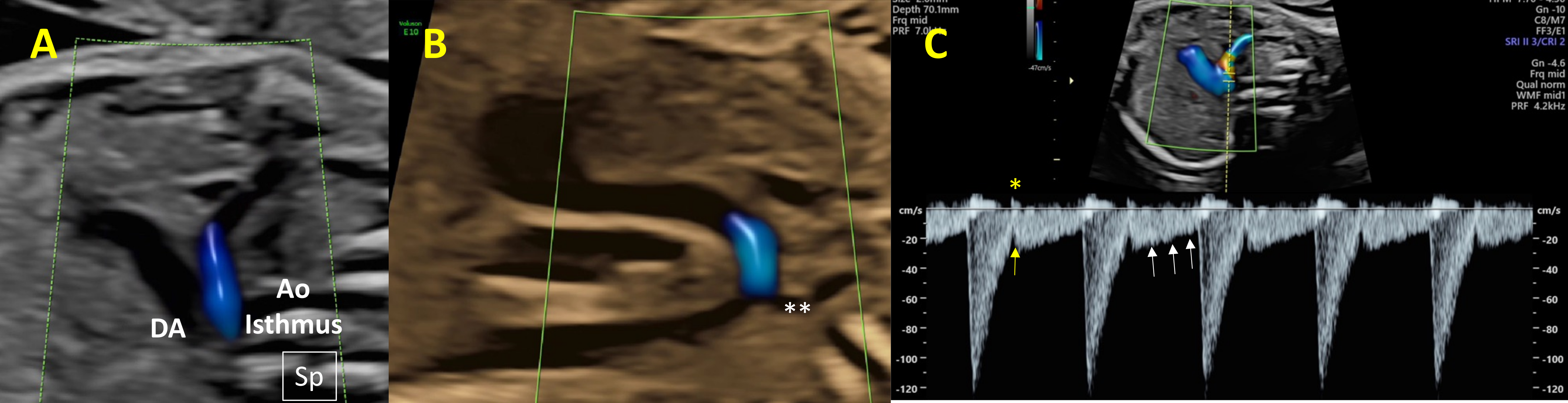

Supplement: S5 Fig — (A) Color Doppler 3-vessel and trachea view showing persistence of diastolic flow in the transverse aortic arch and aortic isthmus compared with the main pulmonary artery and ductus arteriosus. B) Longitudinal aortic arch view evaluated with color Doppler. (C) Aortic isthmus flow characteristics by spectral pulsed-Doppler. Note the presence of a late-systolic notch (yellow arrow) with a small reversal flow wave (*) and clearly increased diastolic flow (white arrows). Ao: aorta, DA: ductus arteriosus, Sp: spine. **Left subclavian artery. (TIFF) [file pcbi.1013096.s010.tiff]
