# Supplementary material for: Understanding the hemodynamic changes in fetuses with coarctation of the aorta using a lumped model of fetal circulation
Source: PLoS Comput Biol. 2025 May 30;21(5):e1013096. doi: 10.1371/journal.pcbi.1013096 (PMC12124859; doi:10.1371/journal.pcbi.1013096)
Supplement: Attachment — Submitted filename: Rebuttal_letter.docx [file pcbi.1013096.s012.docx]

We thank the reviewers for their thoughtful feedback and valuable suggestions.

**Reviewer #1:**

The manuscript applies an established lumped parameter model of the fetal cardiovascular system to explore the impact of geometric perturbations in the aortic isthmus (AoI), which is associated with coarctation of the aorta. The in silico model predicts certain robustness and adaptability of the fetal circulation to this pathophysiological condition and proposes the AoI diastolic velocity as a novel diagnostic indicator.

The authors are established experts in the field, and the manuscript is generally well-written and gives a good example of mechanistic insight into fetal pathophysiology supported by clinical data. Nonetheless, there are a few issues that should be addressed as suggested below.

Firstly, you state (p. 11) that “interactions between cardiovascular components without flow phenomena or wave reflections, [...] was sufficiently accurate for our purposes.”
Aren’t you concerned about the impact of potentially significant pulse wave reflections from the narrowed regions of the aorta? (see Cahill et al. eBioMed. 67, 103326 (2021) 10.1016/j.ebiom.2021.103326). Since the model deals with high Reynolds flows in the complex geometries of the aorta, would it be more appropriate to use distributed flow networks and/or hybrid 0D-1D-3D approaches (for example, Guettouche et al. [9] quoted by the authors, and also Wong et al. Ann. Biomed. Eng. 50, 1158 (2022) 10.1007/s10439-022-02990-5)? Please provide more evidence that these effects can be neglected.

The aorta is a much smaller and more compliant structure ([10.1016/j.avsg.2018.12.060](https://doi.org/10.1016/j.avsg.2018.12.060)) than the umbilical artery, so wave reflections are expected to be dampened. High Reynolds numbers and turbulences are expected for adult CoA, but in the fetus the aorta is much smaller and without a significant stenosis (which develops only neonatally, after ductal closure). Specifically, assuming a normal diameter of 4.7 mm in the aortic isthmus (10.1046/j.1469-0705.2000.00068.x) and a maximum velocity of 75 cm/s (our simulated velocity in the healthy case), we obtain a Reynolds number of 1033. As it is lower than 2300, flow can be considered laminar. In CoA, since the aorta is narrowed and the Reynolds number is directly proportional to the diameter, a much lower Reynolds number is obtained.

Therefore, we did not think a higher dimensional or hybrid model was necessary, but we have added these reasons to the discussion (pages 20-21, lines 421-427):

*“0D models have some limitations. Firstly, 0D lumped models do not consider wave reflections or spatial variation of variables (pressure, volumetric flow rate, and volume) within a component. However, in our study we were interested in the overall distribution of flow, and there were low Reynolds numbers, which support our assumption of laminar flow. Additionally, while the 0D lumped model did not allow for a precise assessment of localized wall shear stresses across the fetal circulation, our approximation was sufficient for analyzing proportional changes relative to the healthy scenario. Therefore, we believe that a 0D lumped model was sufficiently accurate for our purposes.”*

Secondly, I am a bit surprised that the authors did not consult a highly relevant 2015 study of coarctation biomechanics by Zahra Keshavarz-Motamed and others (J. Biomech. 48, 4229,
10.1016/j.jbiomech.2015.10.017). In particular, I invite you to discuss Keshavarz-Motamed et al.’s observation that the trans-coarctation pressure drop is highly sensitive to compliance as well as cardiac output. Thus, the AoI geometry alone could be insufficient to explain the mechanisms of this condition.

We appreciate the valuable contribution of Zahra Keshavarz-Motamed, where they studied the trans-coarctation pressure drop in a pediatric/adult aortic coarctation with an age > 4 years old. This population is very different from ours. While we study a fetal population, with a fully open ductus, no hemodynamic stenosis in the isthmus, and normal vessel wall properties, the population of Keshavarz-Motamed has mostly a closed ductus, a hemodynamic significant stenosis (which is however not tight enough so that it allowed survival up to at least 4 years), and altered wall properties due to longstanding severe proximal hypertension. In the fetal stage, no trans-coarctation pressure drop occurs because the ductus arteriosus, which connects to the descending aorta after the aortic isthmus, equalizes the pressure in the left and right ventricle ([10.1080/07853890.2021.1921253](https://doi.org/10.1080/07853890.2021.1921253)), and thus before and after the coarctation. Additionally, given that there are no abnormal pressures in any of the vessels due to this unique fetal circulation, no changes in vessel compliance have been reported. Also, the dependence on cardiac output is only relevant after birth, given that the ventricular imbalance (of size and stroke volumes) present in the fetal stage compensates any alterations. We have changed the introduction to mention these effects and to further clarify the difference between fetal and adult coarctations (page 4, lines 66-75):

*“Postnatally, after ductal closure, a trans-coarctation pressure gradient is developed [3], and the CoA impairs left ventricular (LV) blood flow to the lower body. This increases afterload and pressure, potentially causing severe cardiac failure[4], and that will also alter vessel wall properties if left untreated. Together with poor lower body perfusion, this becomes an important cause of morbidity and mortality. However, a prenatally CoA can rebalance the circulation due to the presence of the ductus arteriosus (DA). This shunt directs most of the right ventricular (RV) blood toward the aorta, bypassing the CoA site to reach the lower body and return blood to the placenta[2,5], thus preventing the formation of a hemodynamically important stenosis and a trans-coarctation pressure gradient.”*

Finally, although the model is based on prior work by the authors, I would expect further details on the model’s sensitivity to key parameters and comments on the associated uncertainty (please see examples in Marquis Math. Biosci. 304, 9 (2018) 10.1016/j.mbs.2018.07.001, and Mihaela J. R. Soc. Interface. 1720200886 (2020) 10.1098/rsif.2020.0886). This would increase the confidence in the model’s predictions and minimize the risk of overfitting to limited data.

We agree that a sensitivity and uncertainty analysis of our model's parameters would increase the reliability of our predictions. Therefore, we conducted a parametric analysis and included it in the supporting information (Appendix S1, Fig S3). We created 10 distinct parameter sets by applying random variations of up to 10% to the original parameters to generate all CoA scenarios. For each case, we identified its physiological regions (combinations of aortic isthmus narrowing, right/left ventricular end-diastolic volume ratio, and ductus arteriosus dilation, considering changes below 5%, 10%, and 15% across all anatomical sites). We then determined the 25% and 75% confidence interval regions, which closely surrounded or fell within our original regions. This further reinforces our conclusion that the co-occurrence of ventricular disproportion and ductus arteriosus dilation is crucial for compensating for the coarctation. We have added to the discussion (pages 19, lines 387-389):

“*Additionally, the data remained within confidence intervals (see Appendix S1, Fig S3), further reinforcing the validity of our defined physiological regions while accounting for the expected variability within a population.*”

Specific points

The Figures should be more clearly annotated, and the order could be confusing. For example, starting with Figure 4 and indicating the model parameters, which are being fitted and/or perturbed, would put the rest of the paper into proper context for the readership of this journal.

We totally agree that showing the scheme for the fetal model first would improve the understanding of the paper. Therefore, and as we believe our methodology is important for a better understanding of the results, we have reorganized the sections placing Materials and Methods after the Introduction and before the Results.

Figure 1 caption should be extended to explain how the baseline model was parametrized. Considering large variation in the measured control group, it would help to add a mean curve and shaded confidence intervals to aid the assessment. Furthermore, could you factor in the expected dispersion in the normal population when discussing the effect of altered aortic geometry and other CV parameters in Figs 2 & 3?

We have added confidence intervals to Fig 1 (now Fig 2) and how the model was parametrized to its caption (saying it was manually adjusted to ensure the simulation was close to the confidence intervals).

We acknowledge that there are inherent errors when measuring in fetal images, as well as the patient variability that introduces uncertainties into our simulation results. During our study, we have assumed that right/left ventricular depth is maintained in cases of CoA. To validate our results, we conducted further experiments 1) assuming that ventricular depth varies according to ventricular area disproportion (Appendix S1, Fig S1), and 2) generating 10 random variations of our parameters (Appendix S1, Fig S3), accounting for patient variability. Since these additional analyses produced results consistent with our original assumptions, with real data mapping closely to physiological regions in our plots, we concluded that our findings are robust.

We have added to the discussion of Figs 2 and 3, now Figs 3 and 4 (page 19, lines 384-389):

*“While fetal imaging measurements inherently contain some error, the real data still were located within or very close to physiological regions when accounting for ventricular depth variations proportional to ventricular area changes (see Appendix S1, Fig S1). Additionally, the data remained within confidence intervals (see Appendix S1, Fig S3), further reinforcing the validity of our defined physiological regions while accounting for the expected variability within a population.”*

Page 11, l. 212. Please clarify what you mean by “blood pressure velocities”? There is no wave speed present in your model as far as I am aware.

Thank you, this was a typographic error. It should state “blood pressures and velocities”, since we are referring to our results in Fig 3 (now Fig 4).

Figure S3 (c) suggests that both min and max velocities are elevated. Does your model predict that the PI or RI indices are similar to the reference control? If so, what dimensionless index would you propose to detect this condition? Please also see the general point 2 above, which refers to the driving pressure gradient.

In our model, we obtain that the aortic isthmus’ RI is maintained through coarctation while its PI decreases due to the increase in mean velocity. However, no evidence can be found in literature since 1) aortic isthmus Doppler is inaccurate and challenging to obtain in clinical practice, especially when it is coarcted and smaller than usual (10.1016/j.semperi.2022.151584); 2) there is a lot of variability in the velocity patterns among patients (PMID: [24753914](https://pubmed.ncbi.nlm.nih.gov/24753914/)), as some CoA patients present increased diastolic flow and/or reversed flow while others do not; 3) some CoA patients may present additional defects, such as bicuspid aortic valve, ventricular septal defect, or mitral valve disorders (<https://doi.org/10.1161/01.CIR.41.6.1067>). Moreover, the main objective of our study was to understand the physiology of aortic coarctation, not to provide a diagnostic tool, but this would be an interesting topic to investigate in future works, so we have added to the limitations (page 21, lines 440-443):

*“Finally, although our CoA simulations showed increased antegrade diastolic velocity in the aortic isthmus, there is variability in the velocity patterns among patients. Future research should focus on identifying a more reliable diagnostic biomarker for this pathology.”*

I recommend that the codes underpinning the modeled scenarios be made publicly available. Any personally identifiable data should be removed or replaced with aggregated or synthetic illustrations. Sufficient metadata should accompany the codes to allow for the reproduction of key figures and results.

We have publicly released the code and datasets on GitHub (https://github.com/InmaV/CoA_scenarios.git).

**Reviewer #2:**

Villanueva et al. present a study that develops a 0D mathematical model to simulate coarctation of the aorta (CoA) remodeling patterns. The study finds that mild aortic isthmus (AoI) narrowing (80% diameter) requires modest adaptations, while severe narrowing (20% diameter) necessitates significant right ventricular (RV) enlargement and ductus arteriosus (DA) dilation to maintain normal pressures, wall shear stresses, and organ perfusion. Validation with ultrasonographic data supports these findings. Notably, the study identifies specific changes in AoI flow patterns, such as delayed retrograde peaks and increased diastolic velocities, which could enhance early CoA diagnosis. However, despite these compensations, overall fetal hemodynamics, including blood pressure and velocities, remain largely unaltered, except for AoI-specific changes.

The specific comments and questions are:

Discussion:

Line 156: It is observed in Fig. S1 A-B that extreme aortic narrowing could lead to overperfusion of the brain without ventricular disproportion. However, this interpretation may be misleading because Fig. S1 only considers two factors: ventricular disproportion and aortic narrowing. In real CoA patients, other compensatory factors (shown in Fig. 1), such as a dilated ductus arteriosus (DA), can alleviate brain overperfusion by reducing the right ventricular (RV) afterload. This should be acknowledged and discussed further.

In Figure S1 we show the effects of aortic narrowing, ventricular disproportion and ductus arteriosus. We have only chosen these three for creating CoA scenarios since they are the main changes in CoA patients. Our results in Figure S1 A-B show that regardless of the ductus arteriosus dilation, in a severe coarctation (20% of the original isthmus size) only a ventricular disproportion can reduce brain overperfusion and LV pressure. In cases where the combination of ductus arteriosus dilation and ventricular disproportion failed to compensate for aortic narrowing, the cardiovascular system would remodel to ensure adequate perfusion of major organs. However, it is true that very severe coarctations can present additional defects, such as bicuspid aortic valve, ventricular septal defect, or mitral valve disorders (<https://doi.org/10.1161/01.CIR.41.6.1067>), that we have not taken into account, and our database did not contain such severe coarctation to compare with. Therefore, we have added to the discussion (pages 18-19, lines 372-379):

*“Without ventricular disproportion, an extreme aortic narrowing would cause increased intraventricular pressure regardless of DA dilation and potentially harm the heart, leading to overperfusion of the brain and impaired development of the lower body and the placenta (Fig S2 A-B). This is consistent with Giménez et al.[41], who showed that ventricular disproportion normalized LV pressures. However, severe coarctations will probably present additional defects, such as bicuspid aortic valve, ventricular septal defect, or mitral valve disorders [42], that we have not considered in our study since our database only contained isolated CoA.”*

Line 179: The authors emphasize that velocity patterns in the aortic isthmus show a delayed retrograde flow peak in Fig. 3, Row 3. However, on Fig. 1, there is already a phase difference in retrograde flow peaks among real healthy data.

We observed a delayed retrograde flow peak in our simulated CoA scenarios. However, as the reviewer notes, measuring this in clinical practice would be challenging due to data variability. Our primary aim was not to develop a diagnostic tool for CoA, although this could be explored in future work, but rather to characterize its physiological behavior. We have added to the discussion (pages 19-20, lines 403-406):

*“**Additionally, the velocity pattern in AoI showed a delayed retrograde flow peak, possibly due to the longer RV ejection compared to LV, although due to data variability (see Fig 2) accurately measuring this in clinical practice would be challenging.”*

Line 188 and similar instances: The study simulates “extreme CoA” patients using a mathematical model that incorporates many simplifications, such as assuming circular vessel lumens, constant vessel segment diameters, and no significant remodeling of vascular beds. Prior studies indicate that these assumptions may not hold true for extreme CoA cases, particularly given the dynamic remodeling observed in real patients. These limitations should be highlighted more explicitly.

In fetal aortic coarctation, unlike in adults, hemodynamics outside of the isthmus remain compensated due to the presence of fetal shunts (10.1136/hrt.2004.057182), particularly the ductus arteriosus, which redistributes blood from the pulmonary to the systemic circulation without the need to go through the aortic isthmus, thus avoiding elevated pressures. As such, little remodelling has been found. Even in severe cases, the right ventricle (which enlarges as an adaptive mechanism) ensures adequate blood supply to the lower body, while the left ventricle (and the right ventricle in cases of retrograde flow) maintains sufficient cerebral perfusion. As a result, during fetal life, all organs and vessels receive the necessary blood supply, except for the ductus arteriosus, which enlarges, and the aortic isthmus, which is narrowed, meaning no further vascular remodeling is expected. However, it is important to note that severe coarctations are often associated with additional defects, such as ventricular septal defects, which our model did not account for but are now addressed in the Discussion. Significant changes in vascular beds and vessel dimensions are more likely to occur after birth and ductal closure due to the increased afterload and trans-coarctation pressure gradient increase (10.1016/j.ccl.2020.04.003). In this condition, neither the left nor right ventricle can adequately supply the lower body, necessitating further remodeling in mild CoA or potentially causing severe cardiac failure in extreme CoA.

We have clarified the difference between aortic coarctation in fetuses and adults in the introduction (page 4, lines 66-79):

*“Postnatally, after ductal closure, a trans-coarctation pressure gradient is developed [3], and the CoA impairs left ventricular (LV) blood flow to the lower body. This increases afterload and pressure, potentially causing severe cardiac failure[4], and that will also alter vessel wall properties if left untreated. Together with poor lower body perfusion, this becomes an important cause of morbidity and mortality. However, a prenatally CoA can rebalance the circulation due to the presence of the ductus arteriosus (DA). This shunt directs most of the right ventricular (RV) blood toward the aorta, bypassing the CoA site to reach the lower body and return blood to the placenta[2,5], thus preventing the formation of a hemodynamically important stenosis and a trans-coarctation pressure gradient. Additionally, fetuses can often remodel to compensate for adverse circulations. To prevent an increase in LV pressure that would result from forcing blood through the constricted AoI, the fetal cardiovascular system remodels so that most lower body flow is supplied by the RV through the DA[5], ensuring adequate delivery of blood to all organs.*”

Line 203: The authors claim that their model is “adequately accurate” but do not thoroughly address the general lack of accurate wall shear stress assessment in lumped parameter models. A more detailed discussion of this limitation is necessary.

We are using a 0D lumped parameter model, and therefore our wall shear stresses are approximated and do not allow to assess local changes such as in bifurcation. However, since our goal is to assess more global parameters that drive overall vessel growth, without studying localized changes (which would be important e.g. when studying localized dilatation, dissections or atherosclerosis), and we evaluated the changes in wall shear stress between coarctation and a healthy case, rather than calculating the exact value, we think our approximation is sufficient. We have added to the limitations about 0D models (pages 20-21, lines 421-427):

*“0D models have some limitations. Firstly, 0D lumped models do not consider wave reflections or spatial variation of variables (pressure, volumetric flow rate, and volume) within a component. However, in our study we were interested in the overall distribution of flow, and there were low Reynolds numbers, which support our assumption of laminar flow. Additionally, while the 0D lumped model did not allow for a precise assessment of localized wall shear stresses across the fetal circulation, our approximation was sufficient for analyzing proportional changes relative to the healthy scenario. Therefore, we believe that a 0D lumped model was sufficiently accurate for our purposes.”*

Additional Limitation: The study is based on a small cohort, which is a critical limitation, especially considering that CoA is not an exceedingly rare congenital heart defect. A larger dataset would improve the model’s robustness and generalizability.

Our study was based on a small cohort for two main reasons: 1) the limited number of coarctation cases detected during fetal life (10.1016/j.semperi.2022.151584); and 2) of all the cases detected in our hospital, many of them did not have their images stored, so only a few had complete data of sufficient quality to analyse.

We have added to the limitations (page 21, lines 433-437):

*“Furthermore, our study was based on a small cohort, due to the limited number of CoA cases detected at the fetal stage that also had complete, high-quality images available for analysis. Expanding the dataset with a wider range of fetuses at different GA would be beneficial to improve the model’s robustness and generalizability and to allow for direct comparisons between real and simulated CoA velocity traces.”*

Materials and Methods:

Line 253: The assumption that ventricular depths are comparable requires citation. This is crucial to substantiate the validity of this parameter in the model.

Since measuring ventricular depth in a fetus is difficult and, to the best of our knowledge, no studies have indicated that this parameter changes with aortic coarctation, we assumed it to be constant. However, we agree that this assumption needs validation, so we redid our experiments by considering ventricular depth changes proportional to the ventricular area and discussed the results in Appendix S1, Fig S1. We obtained similar results to the original ones, with real data falling within or very close to the physiological regions.

We have added to Materials and Methods (pages 6-7, lines 127-133):

“We computed the RV/LV end-diastolic volume (EDV) ratio using the RV/LV end-diastolic area ratio, assuming that the ventricular depths are comparable, resulting in very similar ratios. This assumption was necessary due to the difficulty of measuring ventricular depth in a fetus and the lack of prior studies indicating its variation. However, to validate this approach, we also conducted experiments considering ventricular depth as proportional to ventricular area variation (see Appendix S1, Fig S1).”

And to the Discussion (page 19, lines 384-387):

“While fetal imaging measurements inherently contain some error, the real data still were located within or very close to physiological regions when accounting for ventricular depth variations proportional to ventricular area changes (see Appendix S1, Fig S1).”

Line 288: The model is based on Pennati et al. (1997) and Garcia-Canadilla's models. However, Pennati’s model is over two decades old, and there have been significant advancements in closed-loop modeling, particularly in heart module development. The authors should justify their choice of an older model over newer approaches.

We have clarified in the methods section that we simulated the heart as the single fiber model described for CircAdapt, and only used Pennati’s and Garcia-Canadilla’s models for the vasculature. Garcia-Canadilla’s study uses an open model, but a highly detailed description of the aortic arch; Pennati’s study provides a very complete description of the fetal cardiovascular system, but a very simple heart (a variable elastance); and CircAdapt includes a more complex heart model, but limited representation of the vasculature. Therefore, used Pennati’s and Garcia-Canadilla’s models for the vasculature, and incorporated the one-fiber heart model from CircAdapt. We added in Materials and Methods (page 8, lines 155-159):

*“Our equivalent electrical lumped model (Fig 1) represented the entire closed-loop circulation without external inputs or outputs. It encompassed the heart, modeled after Arts et al.[24], and the vasculature, based on Pennati et al.[6] and Garcia-Canadilla et al.[7,8], which included the great arteries and all vessels routinely evaluated on fetal ultrasound.”*

Line 289: Since the current model is a composite of several previous models, it is essential to describe it in detail in supplementary materials. This would provide transparency and facilitate reproducibility.

We have clarified in Fig 1 the type of component used in our model to represent the different elements in the cardiovascular system (vascular bed, heart cavity, vessel segment and vessel segment with non-linear resistance). Moreover, all codes have been made publicly available on GitHub (https://github.com/InmaV/CoA_scenarios.git).

Line 297: The non-linear effect described for the AoI compartment should be specified with the corresponding equation to enhance clarity and reproducibility.

We have added the equation in the Materials and Methods section (page 9, lines 178-180):

*“The non-linear term satisfies the equation* $\Delta p\left( t \right)=R\cdot Q\left( t \right)+ K\cdot{Q(t)}^{2}+L\cdot\frac{dQ(t)}{dt}$*, where t is time, p(t) the instantaneous local pressure, Q(t) the volumetric flow rate, R the linear resistance, and L the inductance.”*

Line 310: The middle cerebral artery is described in the text but not depicted in the schematic drawing in Fig. 4. This discrepancy should be addressed for consistency.

We estimated the middle cerebral artery blood flow and pressure from the left and right internal carotids. In the Materials and Methods section, we have defined more clearly how we compute them (pages 9-10, lines 194-199):

*“The middle cerebral artery’s VFR was estimated based on the flow from the left and right internal carotid arteries, assuming that 75% of their combined flow is directed to it[29]. To account for the exclusion of vertebral arteries in our model, a correction factor of 74.3%[30] was applied, representing the total cerebral blood supply provided by the middle cerebral artery. Finally, its pressure was calculated as the mean pressure of the left and right carotid arteries.”*

Line 342: The authors state that compliance is proportional to the square of the diameter, but Garcia’s earlier work (2014) used a cubic relationship. The justification for this change should be provided.

In Garcia’s formulations, compliance is directly proportional to the cube of the vessel radius. However, since it is also inversely proportional to vessel wall thickness, which we assume to be 15% of the radius, the resulting relationship simplifies to a proportionality with the square of the radius. We have clarified this assumption in the Materials and Methods section (page 11, lines 228-231):

*“Using the equations* $R=\frac{8\mu l}{(\pi r^{4})}$*,* $C=\frac{3\pi r^{3}}{(2Eh)}$*,* $L=\frac{\rho l}{\pi r^{2}}$*, where* $r$ *and* $l$ *are the radius and length of the segment,* $h$ *is its wall thickness (assumed to be 15% of* $r$*[14]),* $\mu$ *is blood viscosity and* $E$ *is Young’s modulus, we assumed* $R\propto d^{-4}$*,* $C\propto d^{2}$ *and* $L\propto d^{-2}$*.”*

Line 359: Maximum wall shear stress is calculated using 4μQ/r3, which assumes steady flow conditions. However, fetal circulation is pulsatile, as evidenced by the higher pulsatility indices in CoA patients (Table 1). A wall shear stress equation appropriate for pulsatile conditions should be considered.

The presence of fetal shunts and the adaptive mechanisms of the fetus prevent major changes in Doppler velocity traces (except for the aortic isthmus), since the blood redistribution allows all vessels and organs to be adequately perfused (10.1136/hrt.2004.057182). Therefore, differences between CoA and healthy cases are minimized. While Table 1 shows slightly higher pulsatility indices in CoA cases, the differences were not statistically significant and likely due to the limited sample size in our cohort. Therefore, we did not consider it necessary to apply a wall shear stress equation to account for pulsatile conditions. However, even if pulsatility was present, our analysis focused on proportional changes relative to the healthy scenario, thus any pulsatile effects would not influence our conclusions.

Line 375: The validation of the model against real healthy and CoA cases in Fig. 2 should be elaborated upon, including more detailed results and limitations.

We acknowledge that there are inherent errors when measuring in fetal images, as well as the patient variability that introduces uncertainties into our simulation results. During our study, we have assumed that right/left ventricular depth is maintained in cases of CoA. To validate our results, we conducted further experiments 1) assuming that ventricular depth varies according to ventricular area disproportion (Appendix S1, Fig S1), and 2) generating 10 random variations of our parameters (Appendix S1, Fig S3), accounting for patient variability. Since these additional analyses produced results consistent with our original assumptions, with real data mapping closely to physiological regions in our plots, we concluded that our findings are robust.

We have added to the Discussion of Fig 2, now Fig 3 (page 19, lines 384-389):

*“While fetal imaging measurements inherently contain some error, the real data still were located within or very close to physiological regions when accounting for ventricular depth variations proportional to ventricular area changes (see Appendix S1, Fig S1). Additionally, the data remained within confidence intervals (see Appendix S1, Fig S3), further reinforcing the validity of our defined physiological regions while accounting for the expected variability within a population.”*

Line 383: Details on how ventricular disproportion is simulated are sparse. More information should be provided.

We have now given more details on which parameters we changed to implement ventricular disproportion in Appendix S1.

Since the right/left ventricular end-diastolic volume (RV/LV EDV) ratio is an output in our model, we decreased the LV myocardial mass Vwall. Then, we used a Brent optimization method to increase the RV Vwall maintaining the combined cardiac output. Additionally, to account for the absence of ventricular hypertrophy in CoA cases, we estimated the LV and RV reference areas (Amref, area at zero stress) to preserve the ratio of Vwall to cavity volume in both ventricles. We then decreased the left atrial Vwall and Amref by the same factors as the LV. The mitral and aortic valve orifice areas and the ascending aorta radius were also reduced by factors of 2/3 and 1/3 of the LV Vwall reduction, respectively. Their corresponding resistances, capacitances, and inductances were recalculated as a function of the resulting diameter, assuming $R\propto d^{-4}$, $C\propto d^{2}$ and $L\propto d^{-2}$. Similarly, the right atrium, tricuspid and pulmonary valves, and main pulmonary artery were dilated according to the RV enlargement.

Figures

Figure 1: This figure validates the mathematical model against real healthy data but lacks validation against real CoA data. Models can often replicate the conditions they are based on (e.g., healthy state), but their validity in other states (e.g., CoA) remains uncertain.

The presence of fetal shunts and the adaptive mechanisms of the fetus prevent major changes in Doppler velocity traces (except for the aortic isthmus), since the blood redistribution allows all vessels and organs to be adequately perfused (10.1136/hrt.2004.057182). In our cohort only five cases had aortic isthmus Doppler blood flow velocities measured, each with a different degree of coarctation, making a direct comparison challenging. Therefore, we validated our model using an alternative approach, validating our physiological regions by assessing aortic isthmus and ductus arteriosus diameters, and ventricular size disproportion. We addressed this in Limitations (page 21, lines 433-437):

*“Furthermore, our study was based on a small cohort, due to the limited number of CoA cases detected at the fetal stage that also had complete, high-quality images available for analysis. Expanding the dataset with a wider range of fetuses at different GA would be beneficial to improve the model’s robustness and generalizability and to allow for direct comparisons between real and simulated CoA velocity traces.”*

The control group used for validation spans a gestational age range of 6 weeks (31.8 ± 2.89 weeks), which likely includes significant hemodynamic variability due to fetal growth. Normalization or categorization by gestational age is necessary to improve accuracy.
Even for healthy data, the AoI validation, a central focus of this study, does not match real data adequately.

We reviewed the normal pulsatility index values for fetal vessels across different gestational ages and found minimal variation during a six-week period in the third trimester. Specifically, from 29 to 35 weeks, the pulsatility index decreased only slightly—from 1.08 to 0.94 in the umbilical artery and from 1.99 to 1.86 in the middle cerebral artery (<https://doi>.org/10.1515/jpme.1990.18.3.165). Given this small change, we assumed that gestational age-related differences in hemodynamics were negligible.

We have added to the Discussion section (page 18, lines 361-368):

*“The most notable deviation was observed in the aortic isthmus velocity, which is expected given the challenges in accurately measuring this vessel. Since our model simulates blood flow, converting it to velocity can introduce errors due to uncertainties in both vessel diameter and the spatial flow profile. Moreover, our cases cover a gestational age range of 6 weeks, but considering the small changes in pulsatility indices during the third trimester of pregnancy[36], we assumed that gestational age-related hemodynamic differences were negligible.”*

Figure 4:

The meaning of dashed connections should be clarified. Do they represent specific characteristics or assumptions in the model? The inclusion of specific organs (e.g., kidney, liver, intestines, legs) as separate vascular beds rather than lumping them raises questions. If clinical data were collected for these organs, this should be explicitly stated and validated.

We have clarified the meaning of the dashed connections in the figure’s legend. The kidney, liver, intestines, and legs were included as separate vascular beds because we used Pennati’s model as a reference (<https://doi>.org/10.1016/S1350-4533(97)84634-6). Their study presented these organs as distinct compartments, reported their corresponding resistances and capacitances in a healthy state, and validated these values. Therefore, we used the same structure for the vasculature and scaled their values to represent a fetus at 36 weeks of gestation using the formulas detailed in their paper on parameter scaling (<https://doi>.org/10.1114/1.282).

Additional Comments

Data Sharing: A clear data-sharing plan is missing. To enhance reproducibility and foster collaboration, consider providing anonymized datasets or simulation codes through an open-access platform.

We have now included our data as supplementary material (Datasets S1-S2).

In Appendix S1

Line 9 “Moreover, according to Gallivan et al., W (in grams) and GA (in weeks) in healthy human fetuses are related by log_10(W) = 0.2508 + 0.1458·GA - 0.0016·GA^2.”

Comments:
This equation appears to have been derived from data presented in previous literature. To enhance clarity and credibility, it would be beneficial to include the original data in a table or diagram, alongside the curve fitting obtained in the current study.
Additionally, it seems there may be a typographical error, as “Gallivan et al.” does not appear to be cited properly.

Thank you, the reference for Gallivan’s study was missing. All the formulations in Appendix S1 were not derived by us but by Pennati and Fumero in a separate study, where they described a scaling approach for their model’s parameters throughout gestation (https://doi.org/10.1114/1.282). Their approach also incorporated the relationship between gestational age and estimated fetal weight established by Gallivan. Since we adopted their vascular model, we scaled all parameters from 38 weeks of gestation (the reference values in their original model) to 32 weeks. We have clarified in the Appendix S1 that none of the formulas presented there were developed by us.

Line 19: “Due to their unusual diameter growth, peculiar locations, and functions in the fetal cardiovascular system, the scaling factors of the non-linear resistances in the ductus venosus, ductus arteriosus, and foramen ovale were set to -0.88, -2.5, and -0.6, respectively.”

What is the fluid mechanics rationale for setting the scaling factors of the non-linear resistances in the ductus venosus, ductus arteriosus, and foramen ovale to -0.88, -2.5, and -0.6, respectively?

Pennati and Fumero ((https://doi.org/10.1114/1.282)) reported different scaling factors for the fetal shunts compared to the rest of the vasculature. They justified that the ductus venosus presented a slower diameter growth in the later stages of gestation, which required a lower scaling factor for its diameter. The ductus arteriosus required a lower scaling factor for its non-linear resistance to match clinical Doppler waveforms in the ductus arteriosus and the main pulmonary artery, suggesting that the hydraulic dissipation coefficient in the ductus arteriosus decreases when the vessel size increases. Finally, due to its irregular orifice shape and flexible flap, the foramen ovale required a lower scaling factor to properly model right-to-left ventricular outputs.
